# Suprarenal Extract in Osteomalacia and Rickets

**Published:** 1908-11-21

**Authors:** 


### OBSTETRICS.

### SUPRARENAL EXTRACT IN OSTEOMALACIA AND RICKETS.

The communication made by Professor Bossi of Genoa to the Section of Obstetrics and Gynæcology of the British Medical Association meeting at Sheffield was entitled "The Influence of the Suprarenal Glands on the Bony Skeleton in Relation to Osteomalacia and Rickets." It remains to be seen whether Professor Bossi's suggestions will receive confirmation at the hands of others, but meanwhile little harm could be expected from them, and possibly a great deal of good.

The particular bone condition in which he himself states that he found suprarenal extract so beneficial was osteomalacia. His patient came under his care when she was seven months pregnant in her seventh pregnancy, having begun to develop typical osteomalacia in her fifth pregnancy. The bone trouble had become so extreme that when Professor Bossi saw her she had been absolutely bedridden for four months and her condition was most deplorable. The symptoms were getting worse and worse, the abdomen was becoming more and more distended as the pelvis grew more and more deformed, and day and night the woman was shrieking aloud from pain. Cæsarean section was almost decided upon when experiments suggested the possible utility of suprarenal extract in such a case.

Doses of 0.5 cubic centimetres of the 1 in 1,000 solution of adrenalin were given hypodermically morning and evening. After the injections had been continued for a few days the patient's condition was marvellously changed for the better. At the term of her pregnancy she was spontaneously delivered, and she subsequently became pregnant again and went to term without exhibiting fresh symptoms of osteomalacia.

Professor Bossi allows that there have been failures as well as successes, but he states that both he himself and other physicians have had other cases besides the above that were most happily treated by the same method. It sometimes happened that symptoms of intolerance developed after continuance of the injections for a series of days, but after an intermission of four or five days the treatment could be resumed with the same doses, namely, 0.5, or 1, or even 2 c.c. of the commercial adrenalin solution, 1 in 1,000 twice daily.

He concludes: "I need hardly point out that if these results are confirmed the method will find useful application in the cure of rachitis in children, as one will be able to accelerate the ossification of the skeleton in rickety subjects by the administration of adrenalin."

We think the administration would need to be other than hypodermic if it is to be widely used amongst children, but the suggestion that suprarenal extract may be of use in rickets is worth consideration.

